# Have we increased our efforts to identify strategies which encourage colorectal cancer screening in primary care patients? A review of research outputs over time

**DOI:** 10.1016/j.pmedr.2018.05.015

**Published:** 2018-05-21

**Authors:** Natalie Dodd, Elise Mansfield, Mariko Carey, Christopher Oldmeadow, Rob Sanson-Fisher

**Affiliations:** a Health Behaviour Research Collaborative, School of Medicine and Public Health, Faculty of Health and Medicine, University of Newcastle, Callaghan, NSW, Australia; b Priority Research Centre for Health Behaviour, University of Newcastle, Callaghan, NSW, Australia; c Hunter Medical Research Institute, New Lambton Heights, NSW, Australia; d Clinical Research Design, IT and Statistical Support (CReDITSS), Hunter Medical Research Institute, New Lambton Heights, NSW, Australia

**Keywords:** Review, Colorectal cancer, Early detection of cancer, Primary care, Study design

## Abstract

Globally, colorectal cancer (CRC) screening rates remain suboptimal. Primary care practitioners are supported by clinical practice guidelines which recommend they provide routine CRC screening advice. Published research can provide evidence to improve CRC screening in primary care, however this is dependent on the type and quality of evidence being produced. This review aimed to provide a snapshot of trends in the type and design quality of research reporting CRC screening among primary care patients across three time points: 1993–1995, 2003–2005 and 2013–2015.

Four databases were searched using MeSH headings and keywords. Publications in peer-reviewed journals which reported primary data on CRC screening uptake among primary care patients were eligible for inclusion. Studies meeting eligibility criteria were coded as observational or intervention. Intervention studies were further coded to indicate whether or not they met Effective Practice and Organisation of Care (EPOC) study design criteria.

A total of 102 publications were included. Of these, 65 reported intervention studies and 37 reported observational studies. The proportion of each study type did not change significantly over time. The majority of intervention studies met EPOC design criteria at each time point.

The majority of research in this field has focused on testing strategies to increase CRC screening in primary care patients, as compared to research describing rates of CRC screening in this population. Further research is needed to determine which effective interventions are most likely to be adopted into primary care.

## Highlights

• There was more interventional than descriptive research.
• The majority of interventional research used an EPOC-accepted study design.
• Colorectal cancer screening rates remain low in many cases.
• Research should focus on interventions likely to be adopted into routine practice.

## 1 Introduction

Globally, colorectal cancer (CRC) is the third most diagnosed cancer and the fourth most common cause of cancer death (Ferlay et al., 2013). CRC screening recommendations are reported in clinical practice guidelines in the developed world and include FOBT, sigmoidoscopy and colonoscopy (Australian Cancer Network Colorectal Cancer Guidelines Committee, 2005; European Commission, 2010; U.S. Preventive Services Task Force, 2008). Population-based CRC screening programs are recommended by the World Health Organisation (Wilson & Jungner, 1968) and several developed nations have implemented population-based screening (Benson et al., 2007). Reported CRC screening rates within these programs are suboptimal, ranging from 7% to 68% (Klabunde et al., 2015). This highlights the urgent need to find effective strategies to increase participation in CRC screening. There is increasing interest in the role of primary care providers (PCPs) to encourage participation in screening. Clinical practice guidelines suggest that PCPs provide risk-appropriate CRC screening advice (Australian Cancer Network Colorectal Cancer Guidelines Committee, 2005; European Commission, 2010; Sarfaty, 2008) and PCPs have a high-level of contact with those in the target age range for CRC screening (Britt et al., 2015).

### 1.1 Research type and quality as an indicator of progression of the field

Published research can provide evidence to improve CRC screening in primary care, however this is dependent on the type and quality of evidence being produced. Observational research can provide prevalence data as well as factors associated with an outcome (Theise, 2014). Intervention research that has both internal and external validity can provide data to support causal inferences (Theise, 2014). Exploring the relative effort directed toward observational versus intervention research may help to inform future research directions. For example, if there is a dearth of research of any type, then the field may wish to focus on observational research in order to provide a base for subsequent intervention studies. If there is a predominance of observational research then it may be timely to consider whether efforts would better be focussed on intervention research.

The quality of intervention studies should also be considered. The quality of evidence generated by intervention studies is, in part, determined by the type of experimental design used. The Cochrane Effective Practice and Organisation of Care (EPOC) group specify four study designs which provide robust evidence of effectiveness for interventions: randomised control trials (RCTs), controlled clinical trials (CCTs), interrupted time series (ITS) and controlled before after studies (CBAs) (Cochrane Effective Practice and Organisation of Care Review Group, 2002). Results produced from studies using these designs are less likely to be susceptible to biases, including selection bias and confounding, than those produced from studies using other types of designs (Theise, 2014). While many criteria can be used to comprehensively assess methodological quality, research design provides an initial indicator of research quality.

Clinical practice guidelines report recommendations based on a hierarchy of evidence, with RCTs second only to meta-analyses and systematic reviews (Australian Cancer Network Colorectal Cancer Guidelines Committee, 2005; European Commission, 2010; Guyatt et al., 2015; Royal Australian College of Physicians, 2016). As such it might be expected that the scientific community has increased their research efforts over time from predominantly observational research to high-quality intervention research to inform evidence-based practice.

## 2 Aims

To examine across three time-points (1993–1995, 2003–2005 and 2013–2015), changes in:

• The proportion of observational and intervention research;
• The proportion of intervention studies that used an EPOC-accepted study design.

## 3 Methods

### 3.1 Literature search

Medline, Embase, The Cochrane Library and PSYCINFO databases were searched to identify studies reporting on CRC screening in primary care settings. A start point of 1993 was chosen for the following reasons: 1) Two landmark publications providing evidence that repeated screening with FOBT decreased mortality and that polypectomy via colonoscopy effectively prevented progression of polyps to CRC were published in 1993 (Mandel et al., 1993; Winawer et al., 1993); 2) the earliest mass CRC screening programs commenced in 1992–1993 (Benson et al., 2007). As the purpose of the review was to examine trends over time in the type of research, we examined all relevant publications for three time-points over the past twenty years: 1993–1995 (time point 1), 2003–2005 (time point 2) and 2013–2015 (time point 3).

The following search themes were combined: colorectal cancer, screening and primary care (for full search strategies for each database see Appendix 1). Reference lists of relevant articles were also manually searched to identify additional publications meeting inclusion criteria. The search was limited to include only English language publications and publications with an adult population.

### 3.2 Inclusion and exclusion criteria

All retrieved titles and abstracts were examined for relevance following removal of duplicates.

Publications were eligible for inclusion if they: 1) reported primary data on rates of CRC screening (any form) among primary care patients and used either; a) an observational study design, or; b) an intervention study design where CRC screening was a primary outcome; 2) were conducted either in the primary care setting or using primary care infrastructure/systems, such as electronic patient records; 3) included a sample aged ≥50; 4) were published in a peer-reviewed journal in the years 1993–1995, 2003–2005, 2013–2015; 5) were published in English; 6) had a full manuscript available. Publications that reported on mixed screening for a range of different conditions were included if results for CRC screening were reported separately. Publications that reported on a sample recruited from a variety of settings were included if the outcomes for the primary care sample were reported separately.

Publications were excluded if they: 1) involved participants who had a previous history of CRC, inflammatory bowel disease or those with hereditary disease such as Lynch syndrome or FAP, as people diagnosed with these diseases are at increased risk of CRC when compared to the general population and have differing CRC screening recommendations; 2) reported diagnostic procedures (symptomatic testing); 3) relied on PCP estimates of CRC screening rates; 4) were dissertations, commentaries, book reviews, reports, reviews, case studies, editorials, letters to the editor or conference proceedings.

### 3.3 Data coding

Publication titles and abstracts were initially assessed against the eligibility criteria by one author (ND) and excluded if the study did not meet inclusion criteria. A secondary screen of the abstracts by the same author led to additional publications being excluded. The full texts of the remaining publications were assessed for eligibility. A random subsample of 20% of full text publications were assessed against the inclusion criteria by another author (EM), with any discrepancies resolved via discussion.

All publications meeting the eligibility criteria were categorised according to whether they were: 1) observational studies which reported prevalence of CRC screening among primary care patients; or 2) intervention studies to assess the effectiveness of behavioural interventions to increase CRC screening among primary care patients. Intervention studies were further coded according to whether they met one of the four EPOC design criteria: RCTs, CCTs, CBAs, and ITS.

### 3.4 Analysis

The Kappa statistic was used to assess the level of inter-rater agreement between the authors who assessed the eligibility of full text articles.

To determine changes in proportions of study types over the three time periods we used generalised linear models with a binomial distribution and an identity link. Time was coded as 1, 2 or 3, representing 10 year increments, and assumed to have a linear effect (on the log scale). Coefficients from this model are interpreted as the absolute difference in proportions for each ten year increment in time.

## 4 Results

### 4.1 Search results

**Fig. 1:**
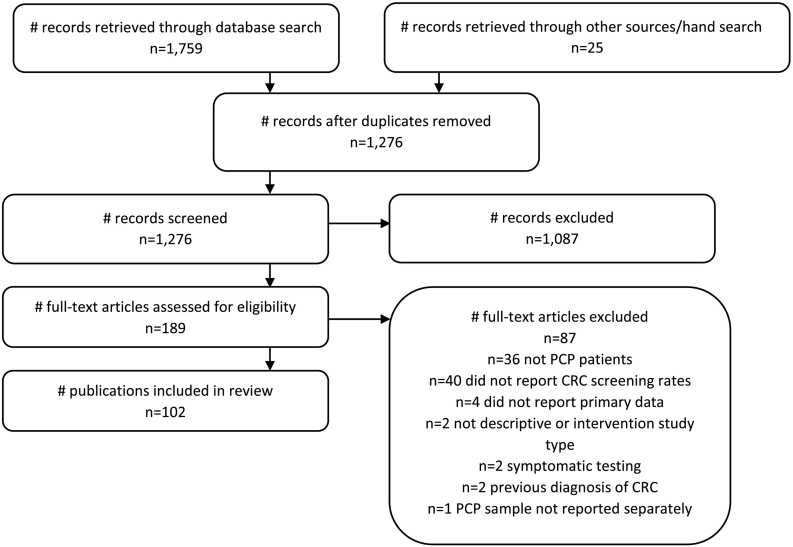
Flow chart of steps and reasons for exclusion.

A total of 1759 publications were retrieved from the searches (see Fig. 1). A further 25 publications were retrieved using a hand search. After duplicates were removed, 1276 publications were assessed against the eligibility criteria. Following initial abstract screening, full-text review was conducted on 189 publications. There were 102 full text publications which met eligibility criteria and were included in the review. The inter-rater agreement between the authors who assessed the eligibility of full text articles was very good (κ = 0.896; 95% CI 0.76–1.0). A full list of included references can be found in Appendix 2.

### 4.2 Changes in proportion of each type of research over time

**Fig. 2:**
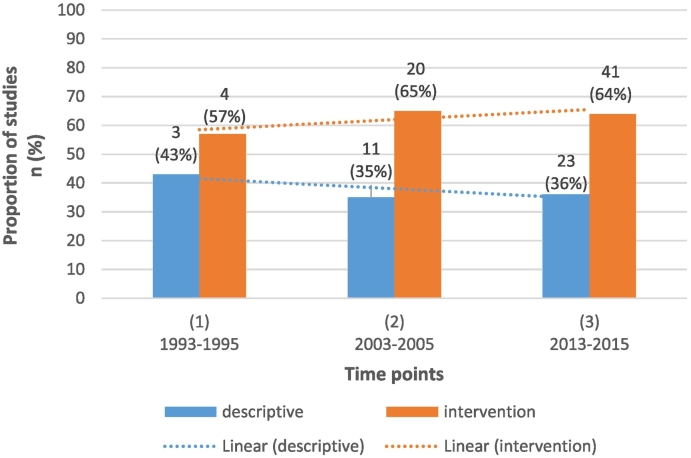
Number and proportion of descriptive and intervention research over time.

Across the time-points, the proportion of studies that utilised an intervention design varied between 57% (time point 1) to 65% (time point 2) (see Fig. 2). The proportion of intervention relative to observational studies did not change significantly over time (risk difference −0.02; 95%CI −0.17–0.13, p = 0.83).

### 4.3 Changes in the proportion of intervention studies that used an EPOC-accepted study design over time

**Fig. 3:**
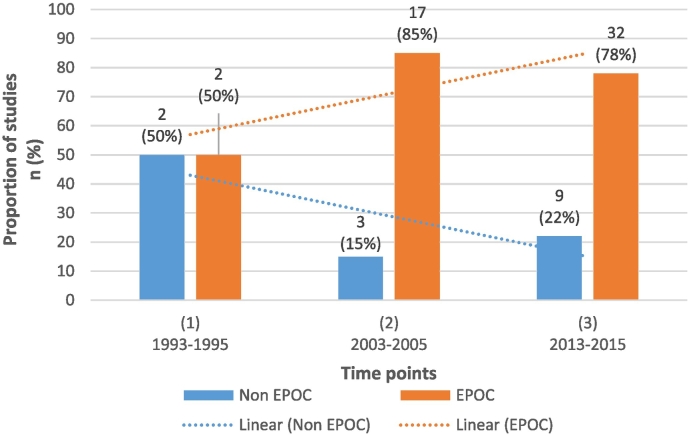
Number and proportion of intervention studies that used an EPOC-accepted study design over time.

At the two most recent time points when the majority (94%) of intervention studies were published, between 78 and 85% of intervention studies used an EPOC-accepted study design. There were no significant changes in the proportion of studies meeting EPOC design criteria across the three time points (see Fig. 3; (risk difference 0.03; 95%CI −0.13–0.20, p = 0.83)).

## 5 Discussion

### 5.1 No statistically significant change in proportion of each type of research over time

The proportion of intervention research was larger than observational research across all time points (approximately 2/3 of studies were intervention at each time point). There was no significant change in the proportion of intervention vs observational research over time. This stands in contrast to previous reviews of the literature on evidence-practice gaps, which have found a higher volume of observational relative to intervention research, and that the proportion of observational research increased over time, relative to intervention research (Bryant et al., 2014; Goyet et al., 2015; Robertson et al., 2015; Mansfield et al., 2016; Waller et al., 2017). Given that intervention studies are often time and resource intensive when compared to observational studies, specifically for CRC screening (Dear et al., 2012) it is encouraging that our results indicate a consistently high proportion of intervention studies being conducted to increase rates of CRC screening.

These findings may reflect a high level of awareness in the field of the need to develop evidence for effective strategies to increase screening uptake, which in turn can lead to improved health outcomes (Mandel et al., 1993; Winawer et al., 1993). National screening registers can provide observational data to describe current patterns of CRC screening behaviours (Klabunde et al., 2015; Australian Institute of Health and Welfare, 2017). However, these data sources are limited in their ability to capture screening which occurs outside of formalised screening programs, such as opportunistic screening in the community or general practice settings. Therefore, observational research remains important in general practice as it can inform the need for development of targeted interventions, and can serve to monitor the impact of changes in policies on current practice. Intervention research in turn, contributes new high-quality data which can be disseminated into practice via clinical practice guidelines (European Commission, 2010; Royal Australian College of Physicians, 2016).

### 5.2 High proportion of intervention studies using EPOC accepted study designs

The majority of intervention studies at the two most recent time points met the EPOC study design criteria (85%, 78% respectively). This high proportion suggests that the intervention research conducted has generally been of high methodological quality. These findings are in contrast to other reviews examining behavioural interventions (grief counselling and smoking cessation), which showed that lower proportions of intervention studies met EPOC design criteria (i.e. 59% (Waller et al., 2015) and 61% (Courtney et al., 2015)). The overall high proportion of intervention studies using EPOC-accepted designs may reflect that primary care settings are amenable to robust study designs such as RCTs due to the available units which can be potentially randomised, including patients, PCPs and practices. It may also reflect a high level of methodological and statistical expertise available in this area, allowing the conduct of high quality intervention trials and consequently the delivery of evidence-based medicine.

## 6 Limitations

These results should be considered in light of several limitations. Firstly, only three time points were included in the analyses. However each time point contained three years (covering 40% of the entire period 1993–2015), providing a reasonable snapshot of research efforts in this field. It is possible that there were extreme year to year variations in research outputs which were not captured by our purposeful sampling approach, leading to incorrect conclusions to be drawn in our review. However, given the range of studies at each time point and the range of three years selected at each time point, this is unlikely to be the case. We only included observational studies which reported the prevalence of screening. Therefore, studies which described attitudes, intentions and the acceptability of screening were omitted. This may have contributed to the lower proportion of observational relative to intervention studies found. Grey literature, including reports, policy documents, dissertations, reviews and protocol papers, were not included in our search. This may have resulted in some relevant studies being missed. As grey literature is not peer-reviewed, its omission may have biased the results toward higher quality studies. In addition, publication bias may limit the extent to which we can rely on publication metrics as a proxy for research effort. Studies with null results may not have been published, leading to an under-representation of the amount of research effort in this area.

## 7 Future directions

While a large proportion of research in this area consisted of high-quality intervention studies, a significant proportion of the population remain under screened for CRC (Navarro et al., 2017). Plateauing rates of CRC screening within some population-based programs (Klabunde et al., 2015) indicate that further research needs to continue exploring the effectiveness of strategies delivered in primary care and other settings in boosting CRC screening participation rates. Observational research indicates that low uptake of CRC screening among primary care patients may be attributable to several barriers, including inadequate time (Aubin-Auger et al., 2011; Guerra et al., 2007; Myers et al., 1999) lack of guideline clarity (Klabunde et al., 2003), lack of patient interest in conversations about CRC screening (Zapka et al., 2011) and cross-cultural issues (Martin et al., 2014). Appropriate primary care-based interventions which overcome these barriers are needed. Systematic reviews of intervention research show that a number of primary care-based strategies are effective in increasing CRC screening uptake (Camilloni et al., 2013; Ferroni et al., 2012; Rawl et al., 2012; Senore et al., 2015), particularly when delivered in conjunction with population-based CRC screening programs (Zajac et al., 2010; Federici et al., 2006; Hewitson et al., 2011; Cole et al., 2002). Multi-factorial systematic interventions have been shown to be most effective in primary care (Sabatino et al., 2012). Despite this research and the importance of the PCP's role in encouraging CRC screening uptake, US primary care studies indicate that only 17% (Malhotra et al., 2014) to 59% (Kern et al., 2014) of primary care patients are screened in accordance with guideline recommendations. Future research should focus particular attention on the feasibility of interventions in practice as well as long-term sustainability. Feasible approaches in this setting may include physician endorsement (Zajac et al., 2010; Hewitson et al., 2011), removal of financial barriers (Potter et al., 2011) and patient education (Senore et al., 2015). Future studies should therefore test these promising strategies using robust experimental designs. Where it is judged that there is sufficient evidence of efficacy for these strategies, studies should then focus on testing ways to effectively implement these into practice using a planned approach which addresses barriers to changing practice, such as stakeholder engagement (community and general practice) and tailoring messages to the target audience (Woolf et al., 2015).

## 8 Conclusion

This review examined trends over time in the proportion of observational and intervention research that explored CRC screening among primary care patients, and the proportion of intervention studies that met EPOC study design criteria. The proportion of intervention research was greater than observational research across all time points, and the proportion of intervention vs observational research did not change over time. The majority of intervention studies used an EPOC-accepted study design, and this proportion did not change across time points. Implementing strategies that use feasible approaches is the next step to embed adoption in primary care and increase CRC screening rates.

The following are the supplementary data related to this article.

Appendix 1 Full search strategy. Appendix 1

Appendix 2 List of included studies. Appendix 2

## Authors' contributions

EM, MC, ND and RSF conceived the study. CO advised on study design, sample size and statistical methods. All authors contributed to the drafting of the manuscript or revising it critically for intellectual content.

## Funding statement

This research has received funding support from the Hunter Cancer Research Alliance Implementation Science Flagship Program as part of the 2017 RHD Student Award initiative, a Strategic Research Partnership Grant (CSR 11–02) from Cancer Council NSW to the Newcastle Cancer Control Collaborative (New-3C) and the Capacity Building Infrastructure Grant Program from the Hunter Medical Research Institute.

A/Prof Carey was supported by a National Health and Medical Research Council ‘Translating Research into Practice Fellowship’ (APP1073031) with co-funding from the Cancer Institute of NSW.

Ms Dodd is supported by The Australian Rotary Health/Rotary District 9650 Bowelscan Funding Partner Scholarship and the MM Sawyer Postgraduate Scholarship in Cancer Research 2014 (G1400854).

The funding bodies financially supported the research, however the responsibility for the study design; collection, management, analysis, and interpretation of data; writing of the report; and the decision to submit the report for publication is that of the authors.

## Declaration of interests

The authors declare that they have no competing interests.
