# Supplementary material for: Have we increased our efforts to identify strategies which encourage colorectal cancer screening in primary care patients? A review of research outputs over time
Source: Prev Med Rep. 2018 May 21;11:100–4. doi: 10.1016/j.pmedr.2018.05.015 (PMC6022456; doi:10.1016/j.pmedr.2018.05.015)
Supplement: Appendix 1 — Full search strategy. [file mmc1.docx]

| **Search** | **Query Medline** |
| --- | --- |
|  | exp Colorectal neoplasms/ |
|  | (colo* cancer* or bowel cancer* or rect* cancer* or sigmoid cancer* or anus cancer* or anal cancer*).tw. |
|  | (colo* neoplas* or bowel neoplas* or rect* neoplas* or sigmoid neoplas* or anal neoplas*).tw. |
|  | (colo* malignanc* or bowel malignanc* or rect* malignanc* or sigmoid malignanc* or anal malignanc*).tw. |
|  | (colo* tumo* or bowel tumo* or rect* tumo* or sigmoid carcinoma* or anal tumo*).tw. |
|  | 1 or 2 or 3 or 4 or 5 |
|  | Mass screening/ or screen*.tw. |
|  | "Early detection of cancer"/ |
|  | Occult blood/ |
|  | (FOBT or "f?ecal occult blood test*").tw. |
|  | ("Guaiac f?ecal occult blood test" or gFOBT).tw. |
|  | ("immuno* f?ecal occult blood test" or iFOBT).tw. |
|  | "f?ecal immuno* test*".tw. |
|  | (Colonoscop* or flexible sigmoidoscop*).tw. |
|  | (stool test* or stool sample or DNA stool).tw. |
|  | exp colonoscopy/ or proctoscopy/ or colonography, computed tomographic/ |
|  | ((early adj3 detect*) or (early adj3 prevent*)). tw. |
|  | 7 or 8 or 9 or 10 or 11 or 12 or 13 or 14 or 15 or 16 or 17 |
|  | general practitioners/ or physicians, family/ or physicians, primary care/ |
|  | exp General Practice/ |
|  | Primary Health Care/ |
|  | ((general or family) adj1 (practice* or practitioner)).tw. |
|  | (primary care or primary healthcare or primary health care or primary health service*).tw. |
|  | 19 or 20 or 21 or 22 or 23 |
|  | "clinical trial".pt. or controlled clinical trial.pt. or "multicenter study".pt. or "randomized controlled trial".pt. or double-blind method/ or exp clinical trials as topic/ or ((randomi?ed adj7 trial*) or (controlled adj3 trial*) or (clinical adj2 trial*) or ((single or doubl* or tripl* or treb*) and (blind* or mask*))).ti,ab,tw. or ("4 arm" or "four arm").ti,ab,tw. |
|  | Intervention Studies/ or evaluation studies/ or evaluation studies as topic/ or program evaluation/ or validation studies as topic/ or ((pre- adj5 post-) or (pretest adj5 posttest) or (program* adj6 evaluat*)).ti,ab. or (effectiveness or intervention).ti,ab. |
|  | 27 or 28 |
|  | **6 and 18 and 24 and 27** |
|  | **limit 28 to (english language and yr="1993 -Current")** |

| **Search** | **Query (Ovid, Embase,)** |
| --- | --- |
|  | exp colon cancer/ or exp colon tumor/ or exp rectum tumor/ |
|  | (colo* cancer* or bowel cancer* or rect* cancer* or sigmoid cancer* or anus cancer* or anal cancer*).tw. |
|  | (colo* neoplas* or bowel neoplas* or rect* neoplas* or sigmoid neoplas* or anal neoplas*).tw. |
|  | (colo* malignanc* or bowel malignanc* or rect* malignanc* or sigmoid malignanc* or anal malignanc*).tw. |
|  | (colo* tumo* or bowel tumo* or rect* tumo* or sigmoid carcinoma* or anal tumo*).tw. |
|  | 1 or 2 or 3 or 4 or 5 |
|  | Mass screening/ or screen*.tw. |
|  | Early diagnosis/ |
|  | Occult blood/ |
|  | (FOBT or "f?ecal occult blood test*").tw. |
|  | ("Guaiac f?ecal occult blood test" or gFOBT).tw. |
|  | ("immuno* f?ecal occult blood test" or iFOBT).tw. |
|  | "f?ecal immuno* test*".tw. |
|  | (Colonoscop* or flexible sigmoidoscop*).tw. |
|  | (stool test* or stool sample or DNA stool).tw. |
|  | colonoscopy/ or computed tomographic colonography / or rectoscopy |
|  | ((early adj3 detect*) or (early adj3 prevent*)). tw. |
|  | 7 or 8 or 9 or 10 or 11 or 12 or 13 or 14 or 15 or 16 or 17 |
|  | general practitioner/ |
|  | General Practice/ |
|  | Primary Health Care/ |
|  | ((general or family) adj1 (practice* or practitioner)).tw. |
|  | (primary care or primary healthcare or primary health care or primary health service*).tw. |
|  | 19 or 20 or 21 or 22 or 23 |
|  | exp "clinical trial (topic)"/ or double blind procedure/ or (clinical trial or randomized controlled trial or controlled clinical trial or multicenter study or phase 1 clinical trial or phase 2 clinical trial or phase 3 clinical trial or phase 4 clinical trial).ct. or ((randomi?ed adj7 trial*) or (controlled adj3 trial*) or (clinical adj2 trial*) or ((single or doubl* or tripl* or treb*) and (blind* or mask*))).ti,ab,tw. or ("4 arm" or "four arm").ti,ab,tw. |
|  | Intervention study/ or evaluation study/ or program evaluation/ or validation study/ or ((pre- adj5 post-) or (pretest adj5 posttest) or (program* adj6 evaluat*)).ti,ab. or (effectiveness or intervention).ti,ab. |
|  | 25 or 26 |
|  | 6 and 18 and 24 and 27 |
|  | **limit 28 to (english language and yr="1993 -Current")** |

| **Search** | **Query (Cochrane)** |
| --- | --- |
|  | MeSH descriptor: [Colorectal Neoplasms] explode all trees |
|  | colo* cancer* or bowel cancer* or rect* cancer* or sigmoid cancer* or anal cancer* |
|  | colo* neoplas* or bowel neoplas* or rect* neoplas* or sigmoid neoplas* or anal neoplas* :ti,ab,kw (Word variations have been searched) |
|  | colo* malignanc* or bowel malignanc* or rect* malignanc* or sigmoid malignanc* or anal malignanc* :ti,ab,kw (Word variations have been searched) |
|  | colo* tumo* or bowel tumo* or rect* tumo* or sigmoid carcinoma* or anal tumo*:ti,ab,kw (Word variations have been searched) |
|  | #1 or #2 or #3 or #4 or #5 |
|  | MeSH descriptor: [Mass Screening] explode all trees |
|  | Screen*:ti,ab,kw (Word variations have been searched) |
|  | MeSH descriptor: [Early Detection of Cancer] explode all trees |
|  | MeSH descriptor: [Occult Blood] explode all trees |
|  | FOBT or "f?ecal occult blood test*" |
|  | "Guaiac f?ecal occult blood test*" or gFOBT |
|  | "immune* f?ecal occult blood test" or iFOBT |
|  | "f?ecal immuno* test*" |
|  | Colonoscop* or flexible sigmoidoscop* |
|  | stool test* or stool sample or DNA stool |
|  | MeSH descriptor: [Colonoscopy] explode all trees |
|  | MeSH descriptor: [Proctoscopy] explode all trees |
|  | (early detect* or early prevent*):ti,ab,kw (Word variations have been searched) |
|  | #7 or #8 or #9 or #10 or #11 or #12 or #13 or #14 or #15 or #16 or #17 or #18 or #19 |
|  | MeSH descriptor: [General Practitioners] explode all trees |
|  | MeSH descriptor: [Physicians, Family] explode all trees |
|  | MeSH descriptor: [Physicians, Primary Care] explode all trees |
|  | MeSH descriptor: [General Practice] explode all trees |
|  | MeSH descriptor: [Primary Health Care] explode all trees |
|  | ((general or family) n/1 (practice* or practitioner*)) |
|  | primary care or primary healthcare or primary health care or primary health service* :ti,ab,kw (Word variations have been searched) |
|  | #21 or #22 or #23 or #24 or #25 or #26 or #27 |
|  | #6 and #20 and #28 |

| **Search** | **Query (Ovid, Psychinfo)** |
| --- | --- |
|  | exp colon disorders/ and neoplasms/ |
|  | (colo* cancer* or bowel cancer* or rect* cancer* or sigmoid cancer* or anus cancer* or anal cancer*).tw. |
|  | (colo* neoplas* or bowel neoplas* or rect* neoplas* or sigmoid neoplas* or anal neoplas*).tw. |
|  | (colo* malignanc* or bowel malignanc* or rect* malignanc* or sigmoid malignanc* or anal malignanc*).tw. |
|  | (colo* tumo* or bowel tumo* or rect* tumo* or sigmoid carcinoma* or anal tumo*).tw. |
|  | 1 or 2 or 3 or 4 or 5 |
|  | Cancer screening/ or screen*.tw. |
|  | ((early adj3 detect*) or (early adj3 prevent*)).tw. |
|  | (FOBT or "f?ecal occult blood test*").tw. |
|  | ("Guaiac f?ecal occult blood test" or gFOBT).tw. |
|  | ("immuno* f?ecal occult blood test" or iFOBT).tw. |
|  | "f?ecal immuno* test*".tw. |
|  | (Colonoscop* or flexible sigmoidoscop*).tw. |
|  | (stool test* or stool sample or DNA stool).tw. |
|  | (colonoscopy* or proctoscopy*).tw. |
|  | ((early adj3 detect*) or (early adj3 prevent*)). tw. |
|  | 7 or 8 or 9 or 10 or 11 or 12 or 13 or 14 or 15 or 16 |
|  | general practitioners/ |
|  | Family medicine/ or family physicians/ |
|  | Primary Health Care/ |
|  | ((general or family) adj1 (practice* or practitioner)).tw. |
|  | (primary care or primary healthcare or primary health care or primary health service*).tw. |
|  | 18 or 19 or 20 or 21 or 22 |
|  | clinical trials/ or "treatment outcome clinical trial".md. or ((randomi?ed adj7 trial*) or ((single or doubl* or tripl* or treb*) and (blind* or mask*)) or (controlled adj3 trial*) or (clinical adj2 trial*)).ti,ab,id. |
|  | program evaluation/ or ((pre- adj5 post-) or (pretest adj5 posttest) or (program* adj6 evaluat*)).ti,ab,id. or (intervention  or effectiveness).ti,ab,id. |
|  | 27 or 28 |
|  | 6 and 17 and 26 |
|  | **limit 27 to (english language and yr="1993 -Current")** |
