# Supplementary material for: Have we increased our efforts to identify strategies which encourage colorectal cancer screening in primary care patients? A review of research outputs over time
Source: Prev Med Rep. 2018 May 21;11:100–4. doi: 10.1016/j.pmedr.2018.05.015 (PMC6022456; doi:10.1016/j.pmedr.2018.05.015)
Supplement: Appendix 2 — List of included studies. [file mmc2.docx]

1. Atlas SJ, Zai AH, Ashburner JM, Chang Y, Percac-Lima S, Levy DE, et al. Non-visit-based cancer screening using a novel population management system. Journal of the American Board of Family Medicine. 2014;27(4):474-85.

2. Barthe J, Perrodeau E, Gilberg S, Ravaud P, Ghasarossian C, Marchand-Buttin F, et al. Impact of a Doctor's Invitation on Participation in Colorectal Cancer Screening: A Cluster Randomized Trial. American Journal of Medicine. 2015;128(9):1024.e1-.e7.

3. Boguradzka A, Wiszniewski M, Kaminski MF, Kraszewska E, Mazurczak-Pluta T, Rzewuska D, et al. The effect of primary care physician counseling on participation rate and use of sedation in colonoscopy-based colorectal cancer screening program--a randomized controlled study. Scand J Gastroenterol. 2014;49(7):878-84.

4. Caffarey SM, Broughton CI, Marks CG. Faecal occult blood screening for colorectal neoplasia in a targeted high-risk population. Br J Surg. 1993;80(11):1399-400.

5. Carney PA, O'Malley JP, Gough A, Buckley DI, Wallace J, Fagnan LJ, et al. Association between documented family history of cancer and screening for breast and colorectal cancer. Prev Med. 2013;57(5):679-84.

6. Casadesus D, Penaloza O, Tewary AM, Moazami D, Simonian A, Goldsmith D. Access matters: Improved detection of premalignant polyps with a screening colonoscopy program for the uninsured. Journal of the National Medical Association. 2015;107(2):46-50.

7. Cavanagh MF, Lane DS, Messina CR, Anderson JC. Clinical case management and navigation for colonoscopy screening in an academic medical center. Cancer. 2013;119(SUPPL. 15):2894-904.

8. Clouston K, Katz A, Martens PJ, Sisler J, Turner D, Lobchuk M, et al. Does access to a colorectal cancer screening website and/or a nurse-managed telephone help line provided to patients by their family physician increase fecal occult blood test uptake?: results from a pragmatic cluster randomized controlled trial. BMC Cancer. 2014;14:263.

9. Cohen-Cline H, Wernli KJ, Bradford SC, Boles-Hall M, Grossman DC. Use of interactive voice response to improve colorectal cancer screening. Medical Care. 2014;52(6):496-9.

10. Corbett M, Chambers SL, Shadbolt B, Hillman LC, Taupin D. Colonoscopy screening for colorectal cancer: The outcomes of two recruitment methods. Medical Journal of Australia. 2004;181(8):423-7.

11. Costanza ME, Luckmann R, Stoddard AM, Avrunin JS, White MJ, Stark JR, et al. Applying a stage model of behavior change to colon cancer screening. Prev Med. 2005;41(3-4):707-19.

12. Crouse A, Sadrzadeh SMH, de Koning L, Naugler C. Sociodemographic correlates of fecal immunotesting for colorectal cancer screening. Clinical Biochemistry. 2015;48(3):105-9.

13. Daly JM, Levy BT, Xu Y. Free fecal immunochemical test disbursement in eight family physician offices. Journal of Community Health: The Publication for Health Promotion and Disease Prevention. 2015;40(5):1008-14.

14. Daly JM, Xu Y, Levy BT. Patients whose physicians recommend colonoscopy and those who follow through. Journal of primary care & community health. 2013;4(2):83-94.

15. Daskalakis C, Vernon SW, Sifri R, DiCarlo M, Cocroft J, Sendecki JA, et al. The effects of test preference, test access, and navigation on colorectal cancer screening. Cancer Epidemiol Biomarkers Prev. 2014;23(8):1521-8.

16. Davis T, Arnold C, Rademaker A, Bennett C, Bailey S, Platt D, et al. Improving colon cancer screening in community clinics. Cancer. 2013;119(21):3879-86.

17. Dietrich AJ, Tobin JN, Robinson CM, Cassells A, Greene MA, Dunn VH, et al. Telephone outreach to increase colon cancer screening in medicaid managed care organizations: a randomized controlled trial. Ann Fam Med. 2013;11(4):335-43.

18. Dignan M, Shelton B, Slone SA, Tolle C, Mohammad S, Schoenberg N, et al. Effectiveness of a primary care practice intervention for increasing colorectal cancer screening in Appalachian Kentucky. Preventive Medicine: An International Journal Devoted to Practice and Theory. 2014;58:70-4.

19. Donahue KE, Reid A, Lefebvre A, Stanek M, Newton WP. Tackling the triple aim in primary care residencies: the I3 POP Collaborative. Family medicine. 2015;47(2):91-7.

20. Farraye FA, Wong M, Hurwitz S, Puleo E, Emmons K, Wallace MB, et al. Barriers to Endoscopic Colorectal Cancer Screening: Are Women Different from Men? American Journal of Gastroenterology. 2004;99(2):341-9.

21. Federici A, Rossi PG, Borgia P, Bartolozzi F, Farchi S, Gausticchi G. The immunochemical faecal occult blood test leads to higher compliance than the guaiac for colorectal cancer screening programmes: A cluster randomized controlled trial. Journal of Medical Screening. 2005;12(2):83-8.

22. Ferreira MR, Dolan NC, Fitzgibbon ML, Davis TC, Gorby N, Ladewski L, et al. Health care provider-directed intervention to increase colorectal cancer screening among veterans: results of a randomized controlled trial. Journal of clinical oncology : official journal of the American Society of Clinical Oncology. 2005;23(7):1548-54.

23. Fischer R, Collet TH, Zeller A, Zimmerli L, Gaspoz JM, Giraudon K, et al. Obesity and overweight associated with lower rates of colorectal cancer screening in Switzerland. European Journal of Cancer Prevention. 2013;22(5):425-30.

24. Fisher DA, Judd L, Sanford NS. Inappropriate colorectal cancer screening: findings and implications. The American journal of gastroenterology. 2005;100(11):2526-30.

25. Freedman JD, Mitchell CK. A simple strategy to improve patient adherence to outpatient fecal occult blood testing. J Gen Intern Med. 1994;9(8):462-4.

26. Giorgi Rossi P, Federici A, Bartolozzi F, Farchi S, Borgia P, Guasticchi G. Trying to improve the compliance to colorectal cancer screening: a complex study design for a complex planning question. Contemp Clin Trials. 2005;26(3):323-30.

27. Goldberg D, Schiff GD, McNutt R, Furumoto-Dawson A, Hammerman M, Hoffman A. Mailings timed to patients' appointments: A controlled trial of fecal occult blood test cards. American Journal of Preventive Medicine. 2004;26(5):431-5.

28. Green BB, Wang CY, Anderson ML, Chubak J, Meenan RT, Vernon SW, et al. Automated intervention with stepped increases in support to increase uptake of colorectal cancer screening: A randomized trial. Annals of Internal Medicine. 2013;158(5 PART 1):301-11.

29. Greiner KA, Daley CM, Epp A, James A, Yeh HW, Geana M, et al. Implementation intentions and colorectal screening: A randomized trial in safety-net clinics. American Journal of Preventive Medicine. 2014;47(6):703-14.

30. Greiner KA, Engelman KK, Hall MA, Ellerbeck EF. Barriers to colorectal cancer screening in rural primary care. Prev Med. 2004;38(3):269-75.

31. Gupta S, Brenner AT, Ratanawongsa N, Inadomi JM. Patient trust in physician influences colorectal cancer screening in low-income patients. American Journal of Preventive Medicine. 2014;47(4):417-23.

32. Hawley ST, Vernon SW, Levin B, Vallejo B. Prevalence of Colorectal Cancer Screening in a Large Medical Organization. Cancer Epidemiology Biomarkers and Prevention. 2004;13(2):314-9.

33. Hayes J, Jackson JL, McNutt GM, Hertz BJ, Ryan JJ, Pawlikowski SA. Association between physician time-unlimited vs time-limited internal medicine board certification and ambulatory patient care quality. JAMA - Journal of the American Medical Association. 2014;312(22):2358-63.

34. Hendren S, Winters P, Humiston S, Idris A, Li SXL, Ford P, et al. Randomized, controlled trial of a multimodal intervention to improve cancer screening rates in a safety-net primary care practice. Journal of General Internal Medicine. 2014;29(1):41-9.

35. Herbert C, Launoy G, Thezee Y, Maurel J, Richir B, Reaud JM, et al. Participants' characteristics in a French colorectal cancer mass screening campaign. Prev Med. 1995;24(5):498-502.

36. Hughes K, Leggett B, Del Mar C, Croese J, Fairley S, Masson J, et al. Guaiac versus immunochemical tests: Faecal occult blood test screening for colorectal cancer in a rural community. Australian and New Zealand Journal of Public Health. 2005;29(4):358-64.

37. Hwang AS, Atlas SJ, Cronin P, Ashburner JM, Shah SJ, He W, et al. Appointment "no-shows" are an independent predictor of subsequent quality of care and resource utilization outcomes. Journal of General Internal Medicine. 2015;30(10):1426-33.

38. Hynam KA, Hart AR, Gay SP, Inglis A, Wicks ACB, Mayberry JF. Screening for colorectal cancer: Reasons for refusal of faecal occult blood testing in a general practice in England. Journal of Epidemiology and Community Health. 1995;49(1):84-6.

39. Jandorf L, Cooperman JL, Stossel LM, Itzkowitz S, Thompson HS, Villagra C, et al. Implementation of culturally targeted patient navigation system for screening colonoscopy in a direct referral system. Health education research. 2013;28(5):803-15.

40. Jandorf L, Gutierrez Y, Lopez J, Christie J, Itzkowitz SH. Use of a patient navigator to increase colorectal cancer screening in an urban neighborhood health clinic. Journal of Urban Health. 2005;82(2):216-24.

41. Jandorf L, Stossel LM, Cooperman JL, Graff Zivin J, Ladabaum U, Hall D, et al. Cost analysis of a patient navigation system to increase screening colonoscopy adherence among urban minorities. Cancer. 2013;119(3):612-20.

42. Jerant A, Kravitz RL, Sohler N, Fiscella K, Romero RL, Parnes B, et al. Sociopsychological tailoring to address colorectal cancer screening disparities: A randomized controlled trial. Annals of Family Medicine. 2014;12(3):204-14.

43. Kern LM, Barron Y, Dhopeshwarkar RV, Edwards A, Kaushal R, Investigators H. Electronic health records and ambulatory quality of care. Journal of General Internal Medicine. 2013;28(4):496-503.

44. Kern LM, Edwards A, Kaushal R. The patient-centered medical home, electronic health records, and quality of care. Annals of Internal Medicine. 2014;160(11):741-9.

45. Kiran T, Wilton AS, Moineddin R, Paszat L, Glazier RH. Effect of payment incentives on cancer screening in Ontario primary care. Annals of Family Medicine. 2014;12(4):317-23.

46. Ko CW, Dominitz JA, Nguyen TD. Fecal occult blood testing in a general medical clinic: Comparison between guaiac-based and immunochemical-based tests. American Journal of Medicine. 2003;115(2):111-4.

47. Krok-Schoen JL, Young GS, Pennell ML, Reiter PL, Katz ML, Post DM, et al. Testing Interventions to Motivate and Educate (TIME): A multi-level intervention to improve colorectal cancer screening. Preventive Medicine Reports. 2015;2:306-13.

48. Kullgren JT, Dicks TN, Fu X, Richardson D, Tzanis GL, Tobi M, et al. Financial incentives for completion of fecal occult blood tests among veterans: a 2-stage, pragmatic, cluster, randomized, controlled trial. Annals of Internal Medicine. 2014;161(10 Suppl):S35-43.

49. Lafata JE, Cooper G, Divine G, Oja-Tebbe N, Flocke SA. Patient-physician colorectal cancer screening discussion content and patients' use of colorectal cancer screening. Patient Education and Counseling. 2014;94(1):76-82.

50. Lafata JE, Williams LK, Ben-Menachem T, Moon C, Divine G. Colorectal carcinoma screening procedure use among primary care patients. Cancer. 2005;104(7):1356-61.

51. Lafata JE, Wunderlich T, Flocke SA, Oja-Tebbe N, Dyer KE, Siminoff LA. Physician use of persuasion and colorectal cancer screening. Translational Behavioral Medicine. 2015;5(1):87-93.

52. Lairson DR, Dicarlo M, Deshmuk AA, Fagan HB, Sifri R, Katurakes N, et al. Cost-effectiveness of a standard intervention versus a navigated intervention on colorectal cancer screening use in primary care. Cancer. 2014;120(7):1042-9.

53. Larkey LK, McClain D, Roe DJ, Hector RD, Lopez AM, Sillanpaa B, et al. Randomized controlled trial of storytelling compared to a personal risk tool intervention on colorectal cancer screening in low-income patients. American journal of health promotion : AJHP. 2015;30(2):e59-e70.

54. Levy BT, Xu Y, Daly JM, Ely JW. A randomized controlled trial to improve colon cancer screening in rural family medicine: An Iowa research network (IRENE) study. Journal of the American Board of Family Medicine. 2013;26(5):486-97.

55. Lofters AK, Ng R, Lobb R. Primary care physician characteristics associated with cancer screening: A retrospective cohort study in Ontario, Canada. Cancer Medicine. 2015;4(2):212-23.

56. Love RR, Baumann LC, Brown RL, Fontana SA, Clark CC, Sanner LA, et al. Cancer prevention services and physician consensus in primary care group practices. Cancer Epidemiology Biomarkers and Prevention. 2004;13(6):958-66.

57. Luckmann R, Costanza ME, Rosal M, White MJ, Cranos C. Referring patients for telephone counseling to promote colorectal cancer screening. American Journal of Managed Care. 2013;19(9):702-8.

58. Malhotra A, Vaughan-Sarrazin M, Charlton ME, Rosenthal GE. Comparison of colorectal cancer screening in veterans based on the location of primary care clinic. Journal of primary care & community health. 2014;5(1):24-9.

59. Markovitz AR, Alexander JA, Lantz PM, Paustian ML. Patient-centered medical home implementation and use of preventive services: the role of practice socioeconomic context. JAMA Intern Med. 2015;175(4):598-606.

60. Matthews BA, Anderson RC, Nattinger AB. Colorectal cancer screening behavior and health insurance status (United States). Cancer causes & control : CCC. 2005;16(6):735-42.

61. Meenan RT, Anderson ML, Chubak J, Vernon SW, Fuller S, Wang C-Y, et al. An economic evaluation of colorectal cancer screening in primary care practice. American Journal of Preventive Medicine. 2015;48(6):714-21.

62. Miller DP, Jr., Kimberly JR, Jr., Case LD, Wofford JL. Using a computer to teach patients about fecal occult blood screening. A randomized trial. J Gen Intern Med. 2005;20(11):984-8.

63. Myers RE, Bittner-Fagan H, Daskalakis C, Sifri R, Vernon SW, Cocroft J, et al. A randomized controlled trial of a tailored navigation and a standard intervention in colorectal cancer screening. Cancer Epidemiology Biomarkers and Prevention. 2013;22(1):109-17.

64. Myers RE, Ruth K, Manne SL, Cocroft J, Sifri R, Ziring B, et al. Effects of genetic and environmental risk assessment feedback on colorectal cancer screening adherence. Journal of Behavioral Medicine. 2015;38(5):777-86.

65. Myers RE, Sifri R, Daskalakis C, DiCarlo M, Geethakumari PR, Cocroft J, et al. Increasing colon cancer screening in primary care among African Americans. Journal of the National Cancer Institute. 2014;106 (12) (no pagination)(dju344).

66. Myers RE, Turner B, Weinberg D, Hyslop T, Hauck WW, Brigham T, et al. Impact of a physician-oriented intervention on follow-up in colorectal cancer screening. Preventive Medicine: An International Journal Devoted to Practice and Theory. 2004;38(4):375-81.

67. Napoles AM, Santoyo-Olsson J, Stewart AL, Olmstead J, Gregorich SE, Farren G, et al. Physician counseling on colorectal cancer screening and receipt of screening among Latino patients. Journal of General Internal Medicine. 2015;30(4):483-9.

68. Nease Jr DE, Green LA. ClinfoTracker: a generalizable prompting tool for primary care. The Journal of the American Board of Family Practice / American Board of Family Practice. 2003;16(2):115-23.

69. Patel P, Forjuoh SN, Avots-Avotins A, Patel T. Identifying opportunities for improved colorectal cancer screening in primary care. Prev Med. 2004;39(2):239-46.

70. Paul CL, Carey ML, Russell G, D'Este C, Sanson-Fisher RW, Zwar N. Prevalence of FOB testing in eastern-Australian general practice patients: what has a national bowel cancer screening program delivered? Health promotion journal of Australia : official journal of Australian Association of Health Promotion Professionals. 2015;26(1):39-44.

71. Payne TH, Galvin M, Taplin S, Austin B, Savarino J, Wagner EH. Practicing population-based care in an HMO: evaluation after 18 months. HMO practice / HMO Group. 1995;9(3):101-6.

72. Percac-Lima S, Lopez L, Ashburner JM, Green AR, Atlas SJ. The longitudinal impact of patient navigation on equity in colorectal cancer screening in a large primary care network. Cancer. 2014;120(13):2025-31.

73. Philip EJ, Shelton RC, Thompson HS, Efuni E, Itzkowitz S, Jandorf L. Is obesity associated with colorectal cancer screening for African American and Latino individuals in the context of patient navigation? Cancer Causes & Control. 2014;25(9):1227-31.

74. Plaskon PP, Fadden MJ. Cancer screening utilization: Is there a role for social work in cancer prevention? Social Work in Health Care. 1995;21(4):59-70.

75. Price-Haywood EG, Harden-Barrios J, Cooper LA. Comparative effectiveness of audit-feedback versus additional physician communication training to improve cancer screening for patients with limited health literacy. Journal of General Internal Medicine. 2014;29(8):1113-21.

76. Ritvo PG, Myers RE, Paszat LF, Tinmouth JM, McColeman J, Mitchell B, et al. Personal navigation increases colorectal cancer screening uptake. Cancer Epidemiology Biomarkers and Prevention. 2015;24(3):506-11.

77. Roetzheim RG, Christman LK, Jacobsen PB, Cantor AB, Schroeder J, Abdulla R, et al. A randomized controlled trial to increase cancer screening among attendees of community health centers. Annals of Family Medicine. 2004;2(4):294-300.

78. Roetzheim RG, Christman LK, Jacobsen PB, Schroeder J, Abdulla R, Hunter S. Long-term results from a randomized controlled trial to increase cancer screening among attendees of Community Health Centers. Annals of Family Medicine. 2005;3(2):109-14.

79. Rosenthal MB, Friedberg MW, Singer SJ, Eastman D, Li Z, Schneider EC. Effect of a multipayer patient-centered medical home on health care utilization and quality: The rhode island chronic care sustainability initiative pilot program. JAMA Intern Med. 2013;173(20):1907-13.

80. Rossi PG, Federici A, Bartolozzi F, Farchi S, Borgia P, Guasticchi G. Understanding non-compliance to colorectal cancer screening: A case control study, nested in a randomised trial [ISRCTN83029072]. BMC Public Health. 2005;5:10P.

81. Ruffin IMT, Gorenflo DW, Murff HJ. Increasing cancer screening rates in primary care: No easy solutions. Journal of Clinical Outcomes Management. 2004;11(12):754-5.

82. Ruffin MTt, Gorenflo DW. Interventions fail to increase cancer screening rates in community-based primary care practices. Prev Med. 2004;39(3):435-40.

83. Saini SD, Vijan S, Schoenfeld P, Powell AA, Moser S, Kerr EA. Role of quality measurement in inappropriate use of screening for colorectal cancer: Retrospective cohort study. BMJ (Online). 2014;348 (no pagination)(g1247).

84. Schapira DV, Pamies RJ, Kumar NB, Herold AH, Van Durme DJ, Woodward LJ, et al. Cancer screening: Knowledge, recommendations, and practices of physicians. Cancer. 1993;71(3):839-43.

85. Schectman G, Barnas G, Laud P, Cantwell L, Horton M, Zarling EJ. Prolonging the return visit interval in primary care. American Journal of Medicine. 2005;118(4):393-9.

86. Scheid DC, Hamm RM, Ramakrishnan K, McCarthy LH, Mold JW, Oklahoma Physicians Resource/Research N. Improving colorectal cancer screening in family medicine: an Oklahoma Physicians Resource/Research Network (OKPRN) study. J Am Board Fam Med. 2013;26(5):498-507.

87. Sewitch MJ, Jiang M, Grad R, Yaffe M, Pavilanis A, Joseph L, et al. Feasibility of a call-in centre to deliver colorectal cancer screening in primary care. Canadian Family Physician. 2013;59(12):e550-e7.

88. Shankleman J, Massat NJ, Khagram L, Ariyanayagam S, Garner A, Khatoon S, et al. Evaluation of a service intervention to improve awareness and uptake of bowel cancer screening in ethnically-diverse areas. British Journal of Cancer. 2014;01.

89. Shaw EK, Ohman-Strickland PA, Piasecki A, Hudson SV, Ferrante JM, McDaniel RR, Jr., et al. Effects of facilitated team meetings and learning collaboratives on colorectal cancer screening rates in primary care practices: A cluster randomized trial. Annals of Family Medicine. 2013;11(3):220-8.

90. Skinner CS, Halm EA, Bishop WP, Ahn C, Gupta S, Farrell D, et al. Impact of Risk Assessment and Tailored versus Nontailored Risk Information on Colorectal Cancer Testing in Primary Care: A Randomized Controlled Trial. Cancer Epidemiol Biomarkers Prev. 2015;24(10):1523-30.

91. Stokamer CL, Tenner CT, Chaudhuri J, Vazquez E, Bini EJ. Randomized Controlled Trial of the Impact of Intensive Patient Education on Compliance with Fecal Occult Blood Testing. Journal of General Internal Medicine. 2005;20(3):278-82.

92. Symonds EL, Pedersen S, Cole SR, Massolino J, Byrne D, Guy J, et al. Improving Participation in Colorectal Cancer Screening: a Randomised Controlled Trial of Sequential Offers of Faecal then Blood Based Non-Invasive Tests. Asian Pacific journal of cancer prevention : APJCP. 2015;16(18):8455-60.

93. Taylor V, Lessler D, Mertens K, Tu SP, Hart A, Chan N, et al. Colorectal cancer screening among African Americans: The importance of physician recommendation. Journal of the National Medical Association. 2003;95(9):806-12.

94. Tinmouth J, Baxter NN, Paszat LF, Rabeneck L, Sutradhar R, Yun L. Using physician-linked mailed invitations in an organised colorectal cancer screening programme: Effectiveness and factors associated with response. BMJ Open. 2014;4 (3) (no pagination)(e004494).

95. Tinmouth J, Patel J, Austin PC, Baxter NN, Brouwers MC, Earle C, et al. Increasing participation in colorectal cancer screening: results from a cluster randomized trial of directly mailed gFOBT kits to previous nonresponders. International journal of cancer. 2015;136(6):E697-703.

96. Tu SP, Chun A, Yasui Y, Kuniyuki A, Yip MP, Taylor V, et al. Adaptation of an evidence-based intervention to promote colorectal cancer screening: a quasi-experimental study. Implementation science : IS. 2014;9:85.

97. Wallace LS, Gupta R. Predictors of screening for breast and colorectal cancer among middle-aged women. Family Medicine. 2003;35(5):349-54.

98. Walsh JM, Salazar R, Terdiman JP, Gildengorin G, Perez-Stable EJ. Promoting use of Colorectal Cancer Screening Tests: Can we change Physician Behavior? Journal of General Internal Medicine. 2005;20(12):1097-101.

99. Weinberg DS, Myers RE, Keenan E, Ruth K, Sifri R, Ziring B, et al. Genetic and environmental risk assessment and colorectal cancer screening in an average-risk population: A randomized trial. Annals of Internal Medicine. 2014;161(8):537-45.

100. Weiss JM, Smith MA, Pickhardt PJ, Kraft SA, Flood GE, Kim DH, et al. Predictors of colorectal cancer screening variation among primary-care providers and clinics. American Journal of Gastroenterology. 2013;108(7):1159-67.

101. You JJ, Liu Y, Kirby J, Vora P, Moayyedi P. Virtual colonoscopy, optical colonoscopy, or fecal occult blood testing for colorectal cancer screening: Results of a pilot randomized controlled trial. Trials. 2015;16 (1) (no pagination)(296).

102. Zapka JG, Lemon SC, Puleo E, Estabrook B, Luckmann R, Erban S. Patient education for colon cancer screening: A randomized trial of a video mailed before a physical examination. Annals of Internal Medicine. 2004;141(9):683-92.
